# Wolff-Parkinson-White Syndrome and Pregnancy: A Case of Intraoperative Supraventricular Tachycardia

**DOI:** 10.7759/cureus.79680

**Published:** 2025-02-26

**Authors:** Hamza Zemrani, Nabil Elachhab, Sophia Lahbabi, Nezha Oudghiri, Rajae Tachinante

**Affiliations:** 1 Maternal Critical Care and Anesthesiology Department, Centre Hospitalo-Universitaire (CHU) Ibn Sina, Rabat, MAR

**Keywords:** emergency caesarean section, intraoperative management, pregnancy, supraventricular tachycardia, wolff-parkinson-white

## Abstract

Wolff-Parkinson-White (WPW) syndrome is a congenital cardiac condition characterized by pre-excitation, which is rarely diagnosed during pregnancy. Nevertheless, during pregnancy, WPW syndrome can lead to potentially fatal arrhythmias in both the mother and fetus, with a low risk of sudden death. It is a ventricular pre-excitation disorder caused by an abnormal conduction pathway that leads to anterograde ventricular activation, bypassing the atrioventricular node. Most anti-arrhythmic drugs pose a threat to the fetus and to the breastfeeding infant, which is why they must be chosen with care. Digoxin is a drug contraindicated in adults with WPW syndrome, and certain drugs such as verapamil increase the risk of ventricular fibrillation. We report the case of a 33-year-old woman, multiparous at 40 weeks, admitted for failure to fully dilate complicated by fetal distress. The patient reported the notion of palpitation since childhood, which was never explored. Intraoperatively, she presented with supraventricular tachycardia requiring care in the operating room.

## Introduction

The first electrocardiogram (ECG) patterns of Wolff-Parkinson-White (WPW) syndrome were first explained in the early 1900s by Frank Wilson and Alfred Wedd. In 1930, Wolff-Parkinson and White described the classic pre-excitation syndrome from a case series of 11 patients presenting with paroxysmal tachycardia in association with ECG changes in sinus rhythm with a short PR interval and a wide QRS complex. Electrocardiographic changes in pre-excitation were first observed in correlation with anatomical evidence of abnormal conductive tissue or shunt pathways in 1943 [1,2]. Characteristic ECG changes, a history of palpitations, chest pain, dyspnea, dizziness, lightheadedness, presyncope, syncope, and collapse help diagnose WPW syndrome [3]. The overall prevalence of WPW syndrome has been estimated at between 0.1% and 0.3% of the population, and the incidence of patients progressing to arrhythmias is around 1%-2% per year. The risk of sudden death is rare, less than 1%, but real. WPW can adversely affect maternal and fetal prognosis. Appropriate maternal diagnosis and management are important to improve maternal and fetal outcomes [4].

## Case presentation

We report the case of a 33-year-old woman, multiparous, on her fifth pregnancy at 40 weeks of amenorrhea, with three live births and one undocumented abortion. The previous three deliveries were by vaginal birth without incident. She was admitted to the operating room for emergency cesarean section for failure to achieve full dilatation and fetal distress. The pre-anesthetic visit noted that the patient reported palpitations unexplored since childhood. Examination found tachycardia at 120 bpm attributed to prolonged labor and pain and blood pressure at 120/70 mmHg. The rest of the examination was normal.

**Figure 1:**
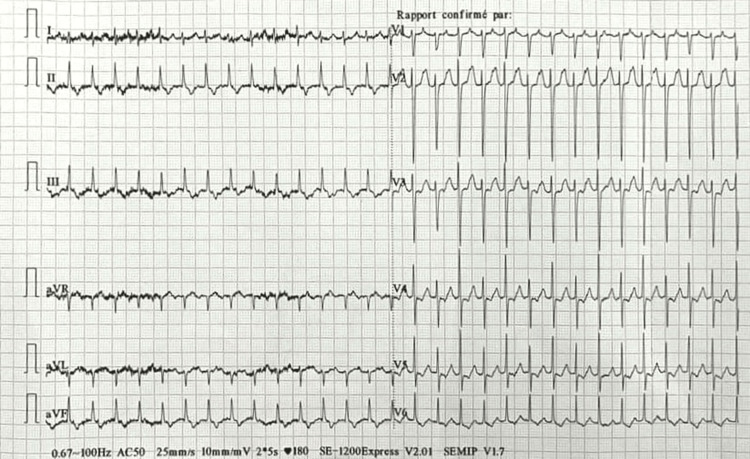
Supraventricular tachycardia

The patient underwent cesarean section under spinal anesthesia with 10 mg of bupivacaine, 25 µg of fentanyl, and 100 µg of morphine. After fetal extraction, the patient manifested supraventricular tachycardia with a mean ventricular rate of 230 bpm and a stable hemodynamic state. She had a BP of 120/70 mmHg with no signs of peripheral hypoperfusion or congestive signs. The electrocardiogram showed supraventricular tachycardia (Figure 1).

**Figure 2:**
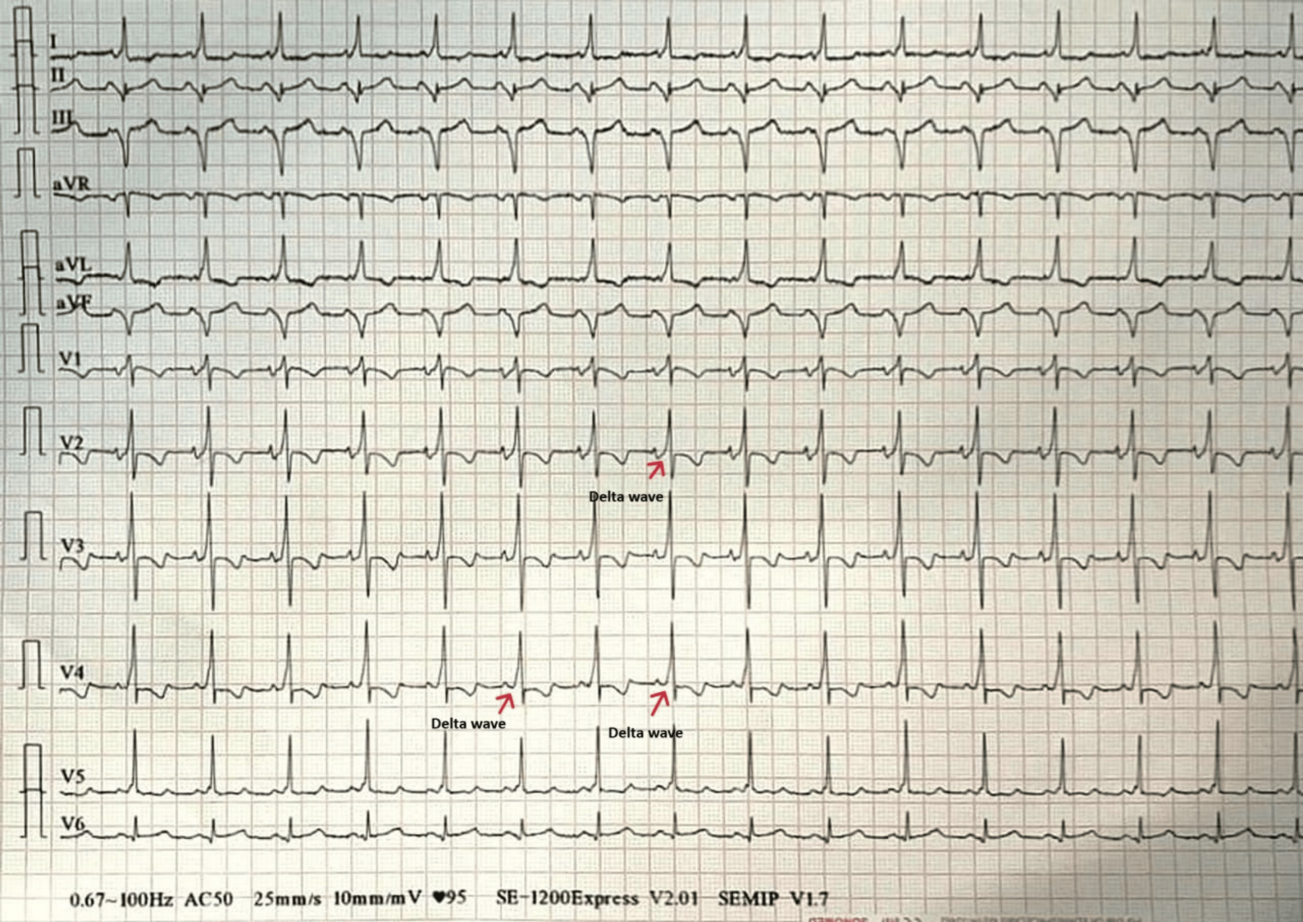
Post-reduction ECG with delta waves (arrows) ECG: electrocardiogram

Treatment was initially based on vagal maneuvers, followed by a loading dose of amiodarone to achieve a heart rate of 180 bpm, and then electrical cardioversion in the absence of verapamil and correction of hypokalemia to 2.6 mEq/L, without success. Tachycardia was subsequently reduced after injection of 4 mg of verapamil. The post-reduction ECG was consistent with WPW with QR serration (delta waves) (Figure 2).

Transthoracic cardiac echocardiography was unremarkable.

Finally, our patient was put on amiodarone maintenance therapy for 24 hours, followed by flecainide 50 mg/12 hours and bisoprolol 2.5 mg/24 hours orally, pending radiofrequency ablation.

## Discussion

In women of childbearing age, the most common arrhythmia is paroxysmal supraventricular tachycardia [5], in which Wolf-Parkinson-White syndrome accounts for the majority of cases, with an incidence of 1.2 per 1,000 [6]. Supraventricular tachycardia in pregnancy is defined as a tachyarrhythmia with a heart rate above 120 bpm [5]. The diagnosis of WPW syndrome is made based on the patient's history and an electrocardiogram (ECG), the latter showing a shortening of the PR interval, delta waves, and widening of the QRS complex [4].

The majority of patients with this syndrome remain asymptomatic. When symptoms do appear, they are usually secondary to tachyarrhythmias such as paroxysmal ventricular tachycardia, atrial fibrillation, atrial flutter, and ventricular fibrillation, which can result in symptoms such as palpitations, dizziness, shortness of breath, syncope, or, in rare cases, sudden death [4].

The exact incidence of WPW syndrome in pregnancy is unknown; however, some reports have indicated that pregnancy may facilitate the development of tachyarrhythmias in patients with asymptomatic pre-excitation.

Maternal blood volume increases by an average of 40% compared with normal levels. The high plasma concentrations of catecholamines, increased sensitivity of adrenergic receptors, and high diastolic volumes associated with pregnancy increase the risk of arrhythmia. An increase in heart rate in these patients may induce unidirectional block in the reentry pathway and trigger reciprocal atrioventricular tachycardia. Changes in conduction physiology and predisposition to complications induced by drugs and techniques used during emergency cesarean section represent an increased risk for both mother and fetus. In addition, stress, anxiety, and fear can activate the sympathetic nervous system, with a potential arrhythmogenic effect [7].

Regional anesthesia offers a significant advantage over general anesthesia as multiple drug administration, laryngoscopic stimulation, intubation, and light planes leading to sympathetic stimulation are avoided. Epidural anesthesia is preferable to spinal anesthesia due to a controlled, segmental block with better hemodynamic stability and postoperative pain management. Avoidance of aorto-caval compression using left lateral tilt helps prevent decreased atrial filling and hence hypotension, reducing the need for vasopressors, which can trigger SVT in patients with WPW syndrome. Phenylephrine has been shown to be effective in treating hypotension without causing an increase in heart rate in these patients [4].

Drugs used during cesarean section can also trigger SVT, e.g., tocolytics and oxytocics. Oxytocin should only be administered as a slow bolus of up to five units or as an intravenous infusion, particularly in the presence of cardiovascular disorders [4].

Our patient was not known to be a WPW carrier and received boluses of ephedrine a few minutes after spinal anesthesia and oxytocin 10 units slow infusion after fetal extraction and 15 minutes before the onset of SVT.

Most anti-arrhythmic drugs cross the placenta and should be considered fetotoxic, and the risk/benefit ratio should be assessed before starting treatment. Anti-arrhythmics should be avoided during the first trimester of pregnancy. Both adenosine and verapamil can prolong the refractory period of the AV node and successfully terminate 90% of acute attacks. Digoxin and calcium channel blockers may accelerate conduction. Beta-blockers are preferred in cases of tachycardia complicating WPW, although they carry the risk of bradycardia, hypoglycemia, intrauterine growth retardation, and apnea in the fetus and newborn. Procainamide is indicated for large-complex tachycardia of uncertain diagnosis. All these medications can be used with caution during pregnancy and breastfeeding [7].

In hemodynamically stable patients, the vagal maneuver can be tried first; if this fails, 6-12 mg of adenosine can be administered intravenously and has a good efficacy and safety profile with an elimination half-life of less than 10 seconds, making it ideally suited to the situation, which has been demonstrated in a number of studies. However, it requires close monitoring of fetal cardiac activity due to the risk of bradycardia. Data on verapamil are limited, but no teratogenic or maternal side effects have been observed. If this fails, intravenous flecainide can be used to terminate the tachycardia [7].

At any stage of pregnancy, an electric shock of 150-200 J is recommended in cases of hemodynamic instability or sustained supraventricular arrhythmia [4]. Fetal heart rate monitoring should be performed before, during, and after cardioversion to assess fetal well-being. If cardioversion is postponed for any reason, drug therapy should be used for 24 hours to see if drug therapy is effective. However, this only increases the duration of maternal tachycardia and the adverse effects of anti-arrhythmic drugs [7].

Prophylactic treatment of arrhythmia includes beta-blockers, and flecainide in the event of intolerance or uncontrollable symptoms [7].

## Conclusions

WPW syndrome in the intrapartum period is a rare and serious condition, requiring special attention as it can be life-threatening for both mother and fetus. The anesthetic procedure must be carefully considered to avoid triggering SVT.

The aim of treatment is to put an end to the tachycardia while respecting the risks to the mother and fetus. The choice of treatment depends on the patient's clinical condition. Electrical cardioversion is reserved for patients with hemodynamic instability and can be used at any stage of pregnancy.

## References

[1] Wolff L, Parkinson J, White PD (2006). Bundle-branch block with short P-R interval in healthy young people prone to paroxysmal tachycardia. 1930. Ann Noninvasive Electrocardiol.

[2] Wilson FN (2002). A case in which the vagus influenced the form of the ventricular complex of the electrocardiogram. 1915. Ann Noninvasive Electrocardiol.

[3] Chhabra L, Goyal A, Benham MD (2025). Wolff-Parkinson-White syndrome. https://pubmed.ncbi.nlm.nih.gov/32119324/.

[4] Palaria U, Rasheed MA, Jain G, Sinha AK (2013). Anesthetic management of Wolff-Parkinson-White syndrome in a pregnant patient posted for emergency caesarean section. Anesth Essays Res.

[5] Nelson-Piercy C (2002). Handbook of obstetric medicine. London: Martin Dunitz.

[6] Oakley C (1997). Heart disease in pregnancy. https://catalog.nlm.nih.gov/discovery/fulldisplay?docid=alma998317583406676&context=L&vid=01NLM_INST:01NLM_INST&lang=en&search_scope=MyInstitution&adaptor=Local%20Search%20Engine&isFrbr=true&tab=LibraryCatalog&query=lds56,contains,Cardiovascular%20Diseases%20--%20complications,AND&mode=advanced&offset=100.

[7] Tan HL, Lie KI (2001). Treatment of tachyarrhythmias during pregnancy and lactation. Eur Heart J.

